# Identification of Novel Gata3 Distal Enhancers Active in Mouse Embryonic Lens

**DOI:** 10.1002/dvdy.24677

**Published:** 2018-11-10

**Authors:** Elena Martynova, Maxime Bouchard, Linda S. Musil, Ales Cvekl

**Affiliations:** ^1^ Departments of Ophthalmology and Visual Sciences and Genetics Albert Einstein College of Medicine Bronx New York; ^2^ Goodman Cancer Research Centre and Department of Biochemistry McGill University Montreal Quebec Canada; ^3^ Department of Biochemistry and Molecular Biology Oregon Health Science University Portland Oregon

**Keywords:** BMP signaling, enhancer, FGF signaling, lens, promoter

## Abstract

Background: The tissue‐specific transcriptional programs during normal development require tight control by distal *cis‐*regulatory elements, such as enhancers, with specific DNA sequences recognized by transcription factors, coactivators, and chromatin remodeling enzymes. Gata3 is a sequence‐specific DNA‐binding transcription factor that regulates formation of multiple tissues and organs, including inner ear, lens, mammary gland, T‐cells, urogenital system, and thyroid gland. In the eye, Gata3 has a highly restricted expression domain in the posterior part of the lens vesicle; however, the underlying regulatory mechanisms are unknown. Results: Here we describe the identification of a novel bipartite Gata3 lens‐specific enhancer located ∼18 kb upstream from its transcriptional start site. We also found that a 5‐kb Gata3 promoter possesses low activity in the lens. The bipartite enhancer contains arrays of AP‐1, Ets‐, and Smad1/5‐binding sites as well as binding sites for lens‐associated DNA‐binding factors. Transient transfection studies of the promoter with the bipartite enhancer showed enhanced activation by BMP4 and FGF2. Conclusions: These studies identify a novel distal enhancer of Gata3 with high activity in lens and indicate that BMP and FGF signaling can up‐regulate expression of Gata3 in differentiating lens fiber cells through the identified Gata3 enhancer and promoter elements. *Developmental Dynamics* 247:1186–1198, 2018. © 2018 The Authors. *Developmental Dynamics* published by Wiley Periodicals, Inc. on behalf of American Association of Anatomists

## Key Findings

- Novel Gata3 lens‐specific enhancer is located 18 kb upstream of transcription start site.
- BMP and FGF signaling can regulate Gata3 expression during mouse lens development.

## Introduction

Precise control of gene expression during development is crucial for establishing hundreds of different cell types forming the embryo, including their spatial organization. Many key developmental genes are regulated by a combination of proximal (i.e., promoter) and distal regulatory elements such as enhancers (Long et al., 2016). Enhancers are defined as relatively short (100–1000 bp) DNA sequences that can activate transcription from one or more cognate promoters over long genomic distances (Schaffner, 2015; Kim and Shiekhattar, 2015). Chromatin of active enhancers is marked by a combination of H3K4me1‐ and H3K27ac‐modified core histone H3 proteins (Long et al., 2016). Individual genes can be regulated by multiple functionally distinct enhancers or closely related “shadow” enhancers. Multiple enhancers are frequently used to direct expression of genes involved in cell type identity and terminal differentiation.

The ocular lens has been extensively employed to study regulation of cell fate decisions, extracellular signaling, and spatial and temporal control of tissue organization, as well as their underlying gene regulatory networks (Cvekl and Zhang, 2017; Cvekl and Ashery‐Padan, 2014; Lang, 2004). At the earliest stage of lens development, the head surface ectoderm thickens and forms a pair of lens placodes that contain lens progenitor cells. Invagination of the lens placode leads to establishment of the three‐dimensional structure referred to as the lens vesicle (Cvekl and Zhang, 2017). At this stage, the cells at the posterior part of the vesicle receive bone morphogenetic protein (BMP) and fibroblast growth factor (FGF) signals from underlying prospective neuroretina, which leads to the polarization of the vesicle and its division into anterior and posterior parts (Lovicu and McAvoy, 2005; Lovicu et al., 2011). Whereas the anterior cells maintain their proliferative capacity and differentiate into the lens epithelium (Zhou et al., 2006; Kallifatidis et al., 2011), the cells at the posterior part withdraw from the cell cycle and differentiate into primary fiber cells that fill the lumen of the lens vesicle (Griep, 2006). Later in development, lens epithelial cells from the germinative zones migrate to equatorial area of the lens (Bassnett and Šikić, 2017), where they undergo cell cycle exit and differentiate into secondary lens fiber cells (Cvekl and Ashery‐Padan, 2014).

The zinc‐finger transcription factor (TF) Gata3 has been extensively studied in multiple systems, including inner ear, kidney, mammary gland, T‐cells, and thyroid gland (Ng et al., 1994; Ho et al., 1991; Ko et al., 1991; Kouros‐Mehr et al., 2006; van der Wees et al., 2004). Gata3 belongs to the GATA family of transcription factors that bind to the consensus sequence (A/T)GATA(A/G) and share a highly conserved DNA‐binding domain (Ko and Engel, 1993; Ko et al., 1991; Merika and Orkin, 1993). A number of tissue‐specific enhancers have been identified within the *Gata3* locus that mediate expression in developing kidney, craniofacial ganglia, T cells and NK cells, embryonic heart, and developing central nervous and urogenital systems (Hosoya‐Ohmura et al., 2011; Hasegawa et al., 2007; Lieuw et al., 1997; Lakshmanan et al., 1999). *Cis*‐regulatory elements important for Gata3 expression in ganglia of peripheral neural system, telencephalon, ribs, ear, and spinal cord lie within 3 kb of the Gata3 transcriptional start site (TSS) (George et al., 1994). Most recent studies have shown a far downstream inner ear enhancer located 571 kbp 3’ to the *Gata3* gene (Moriguchi et al., 2018).

Disruption of Gata3 in mice leads to multiple defects in lens morphogenesis, including dysregulation of cell cycle exit regulators Cdkn1b/p27 and Cdkn1c/p57 and expression of several γ‐crystallins, as well as inhibition of fiber cell differentiation, with retention of nuclei in the presumptive organelle‐free zone (Maeda et al., 2009). Inactivation of Prox1 (Wigle et al., 1999; Audette et al., 2016), Hey1 (Jia et al., 2007), Rbpj (Rowan et al., 2008; Le et al., 2012), and p53 (Wiley et al., 2011) results in a similar spectrum of defects of varying severity, with the most dramatic abnormalities found in Prox1‐null lenses. Despite the importance of Gata3 and Prox1 in lens differentiation, the mechanisms that control transcription of these genes in the lens remain to be established.

Studies of a YAC lacZ reporter transgene in mice demonstrated that eye‐specific enhancer regions are located within 120 kbp of the *Gata3* locus (Lakshmanan et al., 1999). Identification of Gata3 distal enhancer(s) active in the lens is important not only for understanding gene regulatory networks governing lens morphogenesis, but also for a better understanding of how a plethora of enhancers control expression of Gata3 in other tissues and organs. In this study, we first screened potential enhancers based on evolutionarily conserved non‐coding regions and presence of modified histone H3K4me1 in lens chromatin to identify a pair of adjacent ∼650‐bp evolutionarily conserved regions 1A and 1B separated by a ∼1.3‐kbp less‐conserved “linker” that together function as a strong bipartite Gata3 enhancer in vivo in the lens. Additional experiments were conducted to address whether FGF2 and BMP4 activate this enhancer. The results are summarized in a model of the FGF‐ and BMP‐dependent regulation of Gata3 in the differentiating lens fibers.

## Results

### Endogenous Expression of Gata3 During Mouse Lens Development

**Figure 1:**
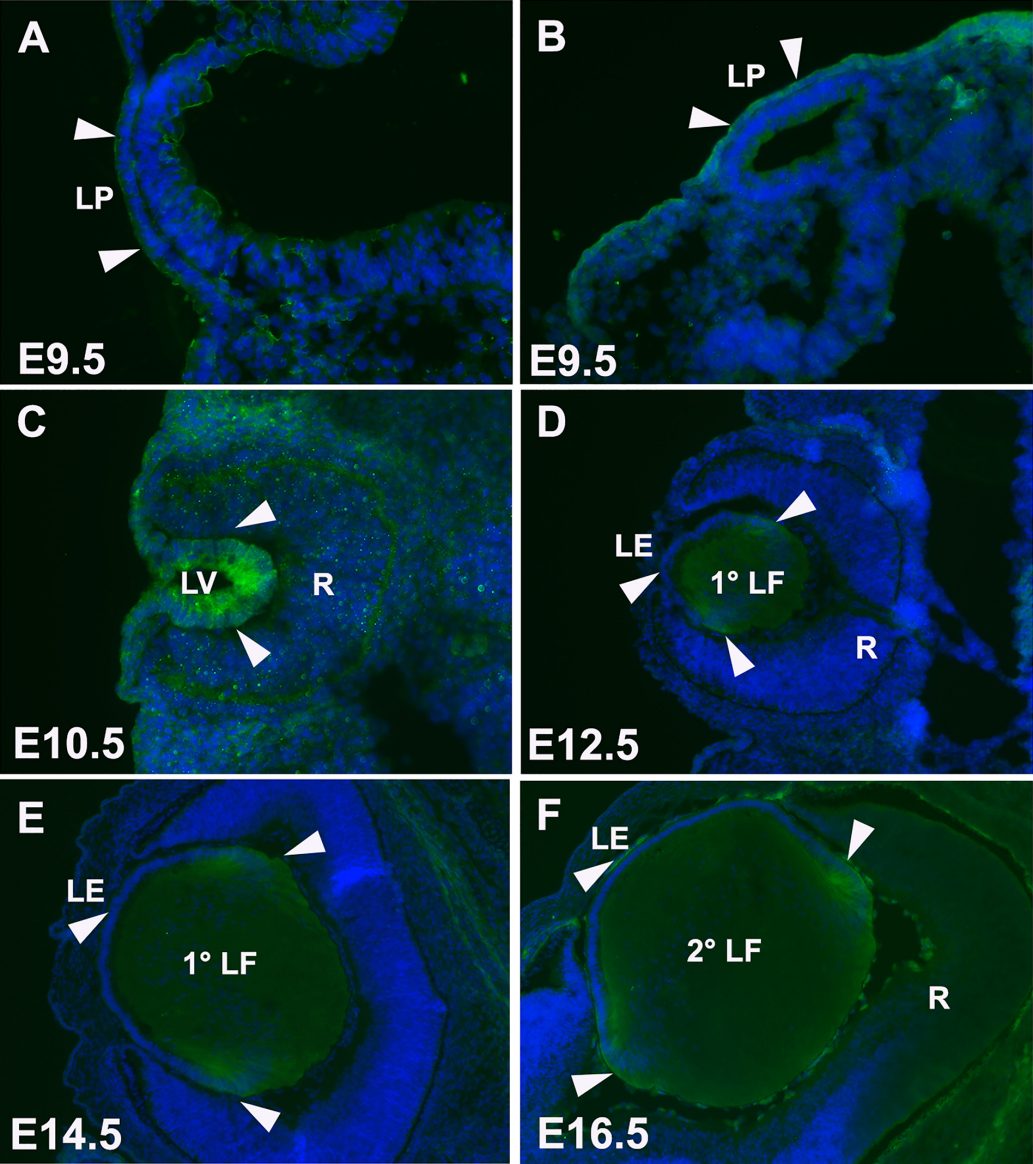
Expression of Gata3 protein during murine lens development. **A–F**: Localization of Gata3 protein at embryonic day (E) 9.5, E10.5, E12.5, E14.5, and E16.5 in mouse lenses from Gata3‐eGFP heterozygous embryos was assessed by immunofluorescence using anti‐eGFP antibody. Anti‐eGFP staining of head surface ectoderm from wild‐type mice was used as a negative control for immunofluorescent staining at E9.5. Nuclei were counterstained with DAPI (blue). White arrowheads in each section indicate Gata3‐eGFP–positive cells. Gata3 expression is observed at lens placode at E9.5 (B), at the posterior part of the lens vesicle at E10.5 (C), and at the transitional area of the lens at E12.5 (D), E14.5 (E), and E16.5 (F). LE, lens epithelium; 1° LF, primary lens fiber cells; LP, lens placode; LV, lens vesicle; OV, optic vesicle; R, retina; 2° LF, secondary lens fiber cells.

Previous studies examined endogenous Gata3 expression during ocular development by in situ hybridization (Lakshmanan et al., 1999) or anti‐Gata3 immunofluorescence microscopy (Maeda et al., 2009). To confirm and extend these studies, we performed immunohistochemical (IHC) analysis of Gata3^GFP/+^ knock‐in mice using anti–green fluorescent protein (GFP) antibody (Grote et al., 2006). We initially detected broad GFP expression at embryonic day (E) 9.5 in the lens preplacodal region as well as in surrounding surface ectoderm (Fig. 1B; negative control is shown in panel A). At E10.5, we found expression in the early lens vesicle (Fig. 1C), confirming the earlier mouse studies using Gata3 antibodies (Maeda et al. 2009). This pattern is also found at the E11.5 lens vesicle (data not shown) (Maeda et al. 2009). At E12.5, when primary lens fiber cells elongate toward the anterior part of the lens, GFP staining was observed in the differentiating lens fiber cells (Fig. 1D). GFP immunoreactivity was detected during E14.5–E16.5 in the transitional area, where cell cycle exit‐coupled differentiation of secondary lens fiber cells occurs (Fig. 1E–F). Gata3 expression was no longer observed at E18.5 or in the neonatal lenses (data not shown), consistent with our recent RNA‐seq studies of mouse embryonic (E14.5, E16.5, and E18.5) and newborn lenses (Zhao et al., 2018). Taken together, our data confirm that in the embryonic lens, Gata3 expression is restricted to the cells that undergo cell cycle exit and lens differentiation. The highest expression is observed at the posterior part of the E10.5 lens vesicle, with earlier expression in the head surface ectoderm consistent with studies of Gata3 in chicken (Sheng and Stern, 1999), mouse (Nardelli et al., 1999) and Zebrafish (Yao et al., 2014).

### Identification of Candidate Gata3 Enhancers In Vitro

**Figure 2:**
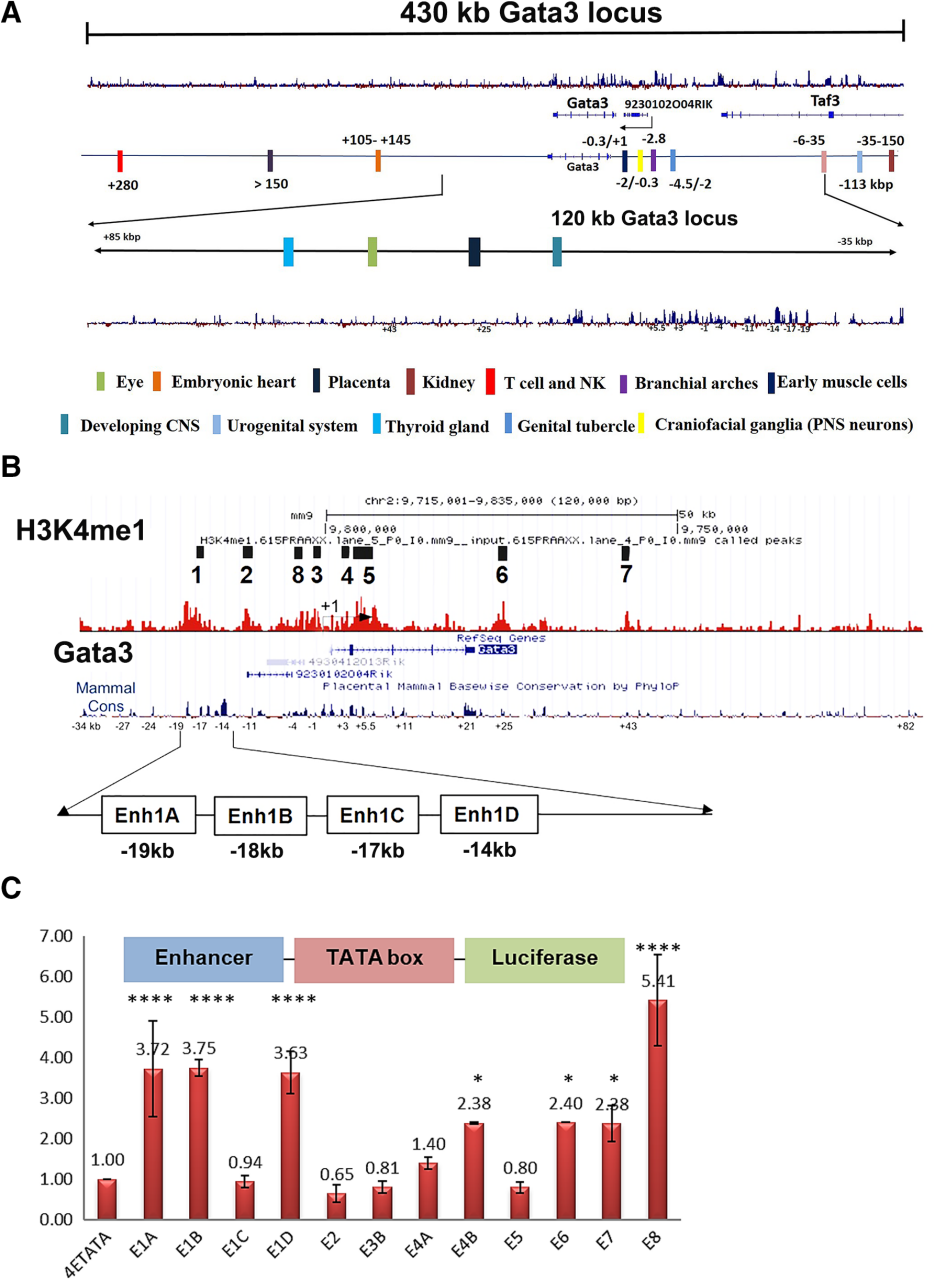
Identification of Gata3 putative regulatory elements **A**: Schematic diagram of the 450‐kb mouse Gata3 locus and its adjacent loci, 9230102O04Rik and Taf3 (chromosome 2). Literature overview revealed various tissue‐specific enhancers for Gata3 located within 450‐kb locus. Regions that regulate Gata3 expression in eye, thyroid gland, developing CNS, and placenta lie within 120‐kb area around the Gata3 TSS. Evolutionary conservation among sets of multiply aligned species was analyzed using UCSC Genome Browser, and conservation tracks are displayed on the diagram. **B**: Analysis of 120‐kb Gata3 locus identified eight putative enhancers that are characterized by high evolutionary conservation and H3K4me1 active enhancer marks. Region 1 contains four distinct peaks for evolutionary conservation and was divided into shorter 1A, 1B, 1C, and 1D regions. Region 4 was subdivided into 4A and 4B regions. **C**: Analysis of 12 putative Gata3 enhancers in dual‐luciferase reporter assay. pGL3‐4ETATA vectors bearing Gata3 candidate regulatory elements were transfected into 293FT cells, and relative enhancer activities from three independent experiments performed in triplicate were calculated by setting the activity of the 4ETATA promoter as 1. The transfection data were normalized using the CMV Renilla luciferase. All experiments were performed in triplicate wells for each condition and repeated three times. Representative data are shown. Significant differences were observed for regions E1A, E1B, E1D, E4B, E6, E7, and E8 (* *P* < 0.01; **** *P* < 0.0001, *t*‐test).

Previous studies reported the use of YAC (Lakshmanan et al., 1999; Zhou et al., 1998) and BAC‐trap (Khandekar et al., 2004) to analyze large genomic regions to localize Gata3 tissue‐specific regulatory elements. Enhancer regions for Gata3 expression in T and NK cells, kidney, developing heart, and other tissues are described elsewhere (Hasegawa et al., 2007; Hosoya‐Ohmura et al., 2011; George et al., 1994; Lakshmanan et al., 1998; Lakshmanan et al., 1999; Lieuw et al., 1997) and reside within the 430‐kb *Gata3* locus (Fig. 2A). It is important to note that the previously identified eye‐specific Gata3 regulatory element(s) are present within a 120‐kb region (‐35 to + 85 kb from TSS) of this locus (Fig. 2A) (Lakshmanan et al., 1999). To identify putative lens‐specific Gata3 enhancers, we analyzed this 120‐kb region and selected 12 candidate sequences that were both highly evolutionary conserved between mammalian species and enriched in H3K4me1‐modified histones (Fig. 2B) (Sun et al., 2015). Note that H3K4me1 region 1 was divided into four shorter evolutionarily conserved regions, 1A–1D; region 4 was divided into 4A and 4B; and region 8 is within the 5‐kb Gata3 “extended” promoter. We next employed a transient transfection assay to test putative Gata3 enhancers in Gata3‐expressing 293T cells using a heterologous TATA promoter that drives luciferase reporter gene expression. We identified enhancer activities in at least five regions, termed E1A (‐19.1/‐19.7 kb), E1B (‐17.1/‐17.7 kb), E1D (‐13.8/‐14.7 kb), E7 (+42.7/+43.5 kb), and E8 (‐3.3/‐4.1 kb) (Fig. 2C).

### Eye‐specific Enhancers are Located 18 kbp Upstream of the Mouse *Gata3* Gene

**Figure 3:**
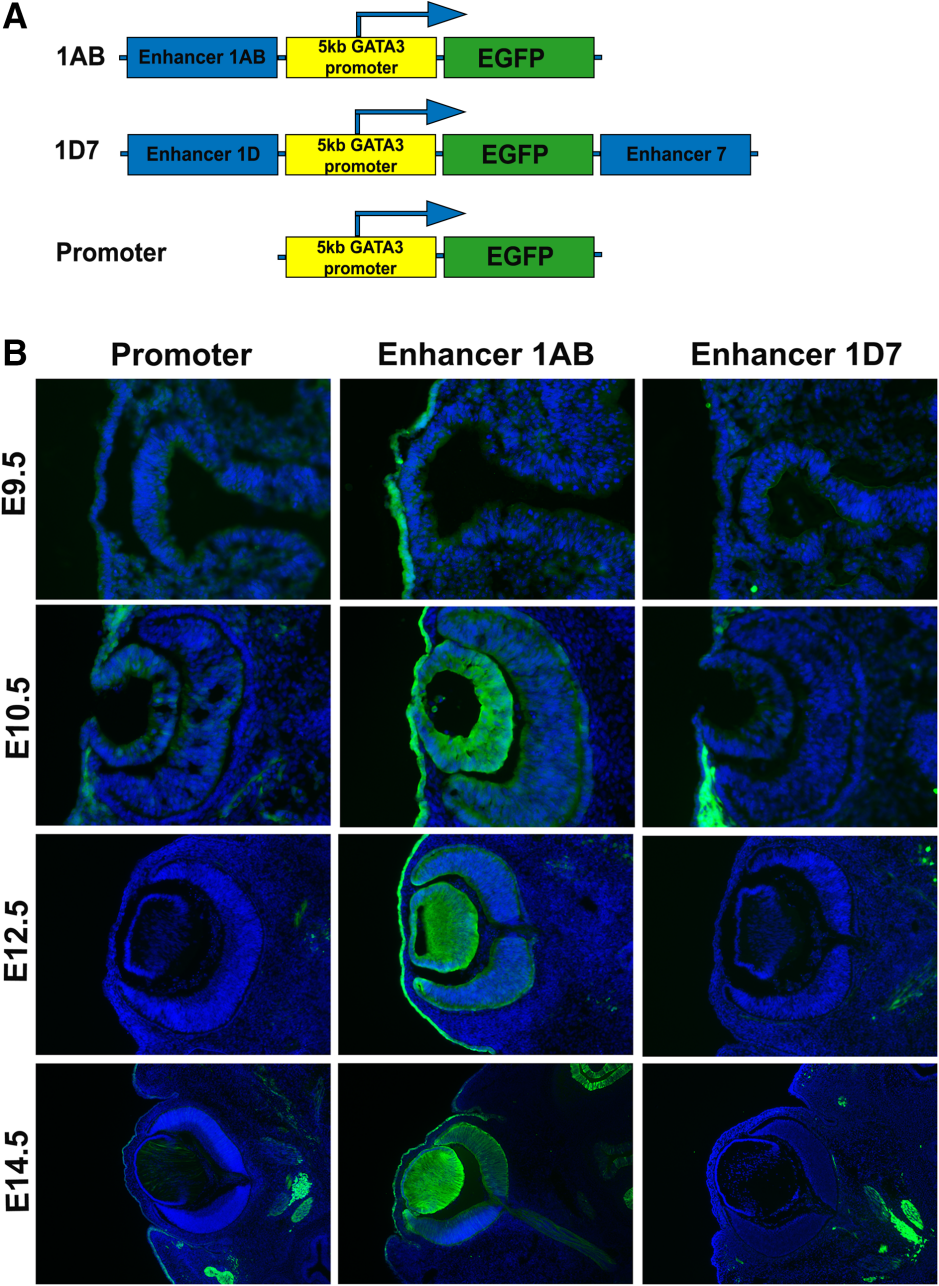
Analysis of Gata3 regulatory elements in vivo. **A**: Schematic representation of three Gata3 transgene constructs. Enhancers 1A and 1B were combined with the 5‐kb Gata3 promoter (1AB construct), enhancers 1D and 7 were combined with 5‐kb Gata3 promoter (1D7 construct), and 5‐kb Gata3 promoter alone generated the PROMOTER line. **B**: Spatial and temporal expression of eGFP driven by three transgenic constructs, PROMOTER, 1AB, and 1D7, in developing lens. Transverse sections of the embryonic eye at E9.5, E10.5, E12.5, and E14.5 were analyzed by immunofluorescence microscopy using anti‐eGFP antibody. The 1AB construct regulates strong eGFP expression in the lens preplacodal region at E9.5, developing lens, cornea, and retina starting at E10.5. eGFP expression is restricted to differentiated lens fiber cells and not to lens epithelial cells at E14.5. PROM construct exhibits weak eGFP staining. Transgene expression is observed in other ectodermal cells outside of the lens for PROM and 1D7 constructs. eGFP is green, nuclei are counterstained with DAPI (blue).

To evaluate the in vivo activity of putative Gata3 enhancers during mouse development, we generated transgenic lines that express eGFP driven by the homologous Gata3 promoter coupled with candidate enhancers. We used an extended 5‐kbp Gata3 promoter (PROMOTER) that also includes enhancer E8 located 4 kbp upstream of TSS. Given the proximal location of enhancers 1A and 1B, their sequences were combined to generate a 1AB/PROMOTER reporter construct (1AB). Enhancers E1D and E7 were tested together as the 1D7/PROMOTER reporter (1D7) (Fig. 3A). The PROMOTER, 1AB, and 1D7 founder mice yielded three, seven, and four independent lines, respectively. Strikingly, all founders of the 1AB line exhibited green eye fluorescence under UV light (data not shown). The copy number variation for selected transgenic lines was determined by quantitative polymerase chain reaction (qPCR) as described in Experimental Procedures, and relative eGFP expressions were measured as intensities of anti‐GFP immunofluorescent stainings by ImageJ software (Table 1).

**Table 1:** Characterization of PROMOTER, 1AB and 1D7 Transgenic Mice Lines

Construct	Regulatory elements	Founders	Predicted CN	Relative eGFP expression
PROMOTER	5 kb Gata3 promoter	PROM1	1	2.1 ± 0.5
		PROM2	2	1.2 ± 0.1
		PROM3	15	1.3 ± 0.1
1AB	1A, 1B enhancers, 5kb Gata3 promoter	1AB1	2	32.8 ± 3.3
		1AB2	2	7.9 ± 2.7
		1AB5	2	10.8 ± 1.6
		1AB6	17	5.9 ± 2.4
1D7	1D, 7 enhancers, 5kb Gata3 promoter	1D7‐1	1	1.1 ± 0.1
		1D7‐2	3	1.2 ± 0.1
		1D7‐3	2	1.2 ± 0.1

Copy number (CN) variation analysis was performed using eGFP TaqMan assay, and mouse Tfrc gene served as a reference. eGFP expression in transgenic lenses was analyzed by immunofluorescence and quantified relative to wild‐type Gata3 (GFP) lens by ImageJ software.

Analysis of eGFP expression during ocular development revealed that all five 1AB lines first expressed the transgene at E9.5 (Fig. 3B), which is consistent with our observations of endogenous Gata3 expression in the preplacodal region of the lens and surrounding surface ectoderm (Fig. 1B). From E10.5 in 1AB embryos, eGFP proteins were detected in the surface ectoderm/prospective corneal epithelium, lens vesicle, inner and outer nuclear layers of the optic cup, and prospective optic nerve (Fig. 3B). Later in development at E12.5 and E14.5, eGFP proteins are expressed in the differentiating lens fiber cells, but not in the proliferating lens epithelial cells at the anterior part of the lens. In contrast, the PROMOTER lines exhibited much weaker activity in the developing lens and cornea (Fig. 3B; Table 1) as well as in adjacent ectodermal tissues and extraocular muscles. The 1D7 lines also showed transgene expression in the neighboring ectodermal tissues and extraocular muscles (Fig. 3B). However, all 1D7 lines were negative in developing ocular tissues. Taken together, these data demonstrate that the combined 1AB regions coupled with the 5‐kb Gata3 promoter drive robust eGFP transgene expressions in the developing lens, cornea, and retina.

### Identification of Conserved *Cis*‐sites and Candidate Transcription Factors that Regulate Gata3 Expression

**Figure 4:**
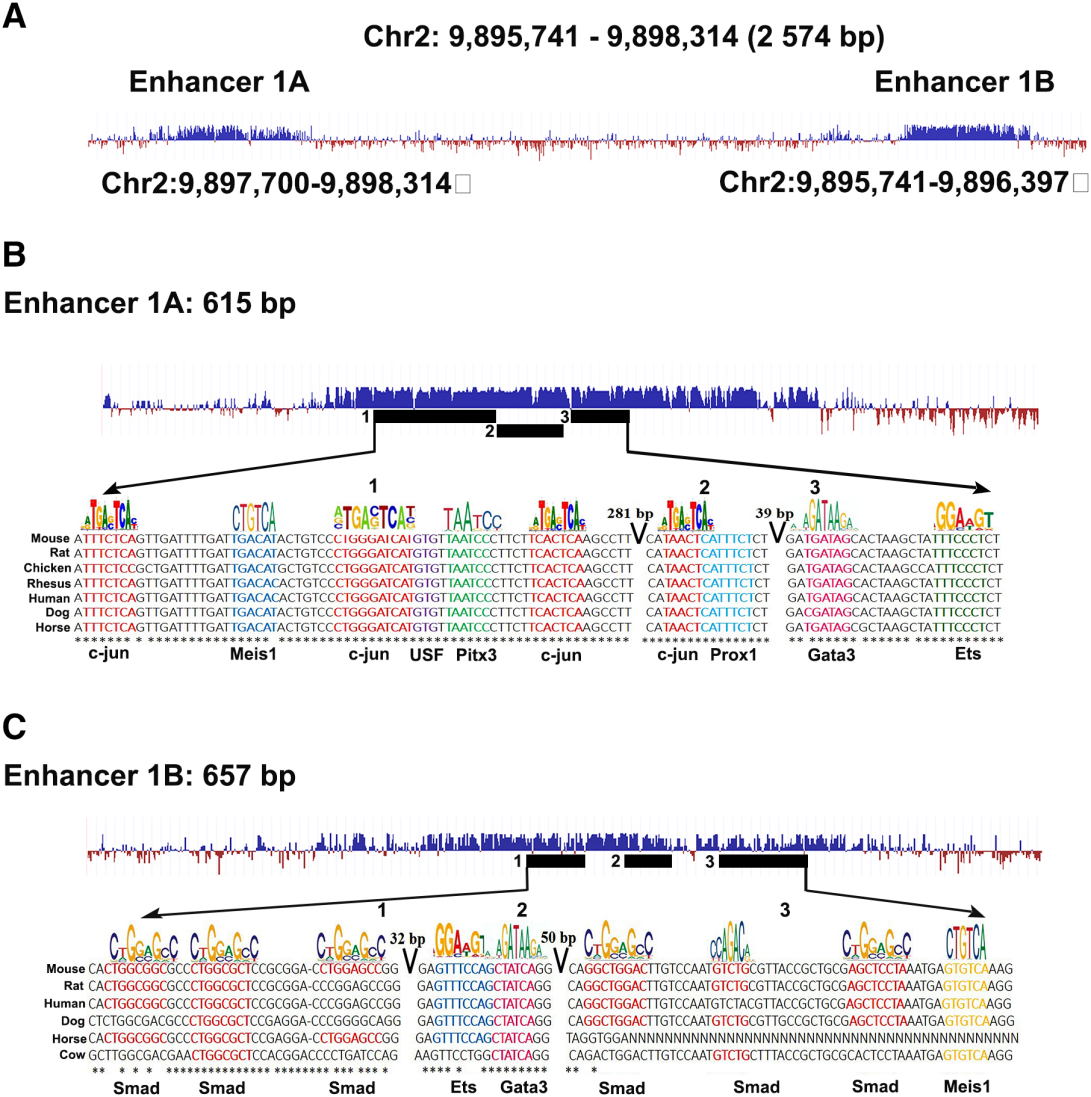
Examination of putative TF binding sites within the lens‐specific Gata3 1A and 1B enhancer regions. **A**: Schematic representation of bipartite 1AB enhancer 18 kb upstream of *Gata3* gene; 2.5‐kb locus includes highly evolutionarily conserved 1A and 1B regions and a linker. **B**: Example of the areas within the 1A regulatory element that are enriched with binding sites for TFs downstream of FGF signaling. **C**: Enhancer 1B displays enrichment of BMP‐regulated Smad motifs and a putative Gata3 binding site.

**Figure 5:**
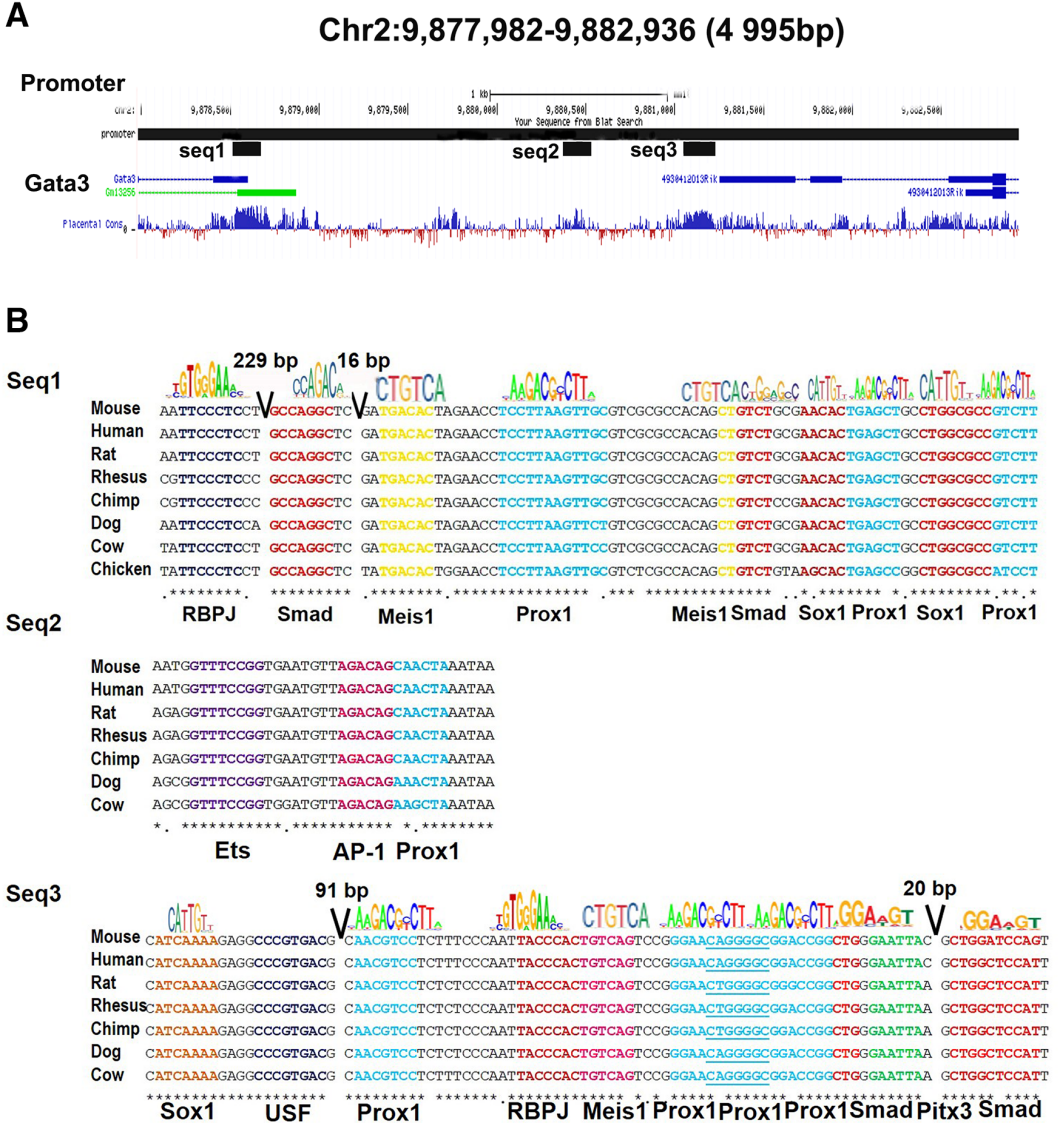
Multiple binding sites for FGF‐ and BMP‐ regulated transcription factors are present in the 5‐kb promoter of the mouse *Gata3* gene. **A**: Schematic representation of the 5‐kb *Gata3* promoter region and its adjacent loci (9230102O04Rik). Evolutionary conservation analysis using VISTA Point tool and UCSC browser identified three highly conserved regions within *Gata3* promoter. **B**: Predicted Smad, Ets, Prox1, RBPJ, Meis1, Sox1, Pitx3, and AP‐1 binding sites within evolutionarily conserved regions of the *Gata3* promoter. The binding sites were searched using Fuzznuc software by allowing up to one mismatch in the consensus sequences.

Transcriptional enhancers are composed of concentrated clusters of transcription factor–binding sites, including signal‐regulated transcription factors (SRTFs) (Barolo and Posakony, 2002; Long et al., 2016). We first compared the sequences of regions 1A and 1B (Fig. 4) and the Gata3 promoter (Fig. 5) in multiple species, including mouse, rat, human, dog, cow, and horse, and identified numerous blocks of evolutionarily conserved sequences. Several binding sites for transcription factors implicated in lens development were found in both enhancer regions, including Gata3, Meis1/2, Pitx3, and Prox1 sites (Fig. 4) (Cvekl and Zhang, 2017). To address which SRTFs are present, we first focused on the FGF‐regulated AP‐1 factor c‐Jun (Xie et al., 2016). It is important to note that both c‐Jun and Gata3 have similar expression domains at the posterior compartment of the lens vesicle at E11.5 and in the transitional area of the lens at E14.5 (Xie et al., 2016; Maeda et al., 2009). It has been shown earlier that Gata3 expression is regulated by BMP signaling in cranial neural crest cells (Bonilla‐Claudio et al., 2012) and hair follicles (Kobielak et al., 2003; Andl et al., 2004). Both FGF and BMP signaling regulate lens fiber cell differentiation (Jarrin et al., 2012; Boswell et al., 2008; Boswell and Musil, 2015). Thus, BMP‐regulated Smad binding sites were also included in the analysis. We found that enhancer 1A contained multiple AP‐1 and Ets‐sites, together with sites for Prox1, Pitx3, and Gata3 itself (Fig. 4B). In contrast, enhancer 1B contains multiple Smad‐binding sites, along with Gata3, Ets and Meis1 motifs (Fig. 4C). Arrays of FGF‐ and BMP‐ regulated binding motifs recognized by SRTFs were found at three evolutionary conserved regions within the 5‐kbp Gata3 promoter (Fig. 5). We conclude that all three Gata3 regulatory regions required for lens expression comprise multiple binding sites for lens transcriptional regulators, including specific SRTF downstream of BMP and FGF signaling.

### Characterization of Individual Enhancers 1A and 1B

**Figure 6:**
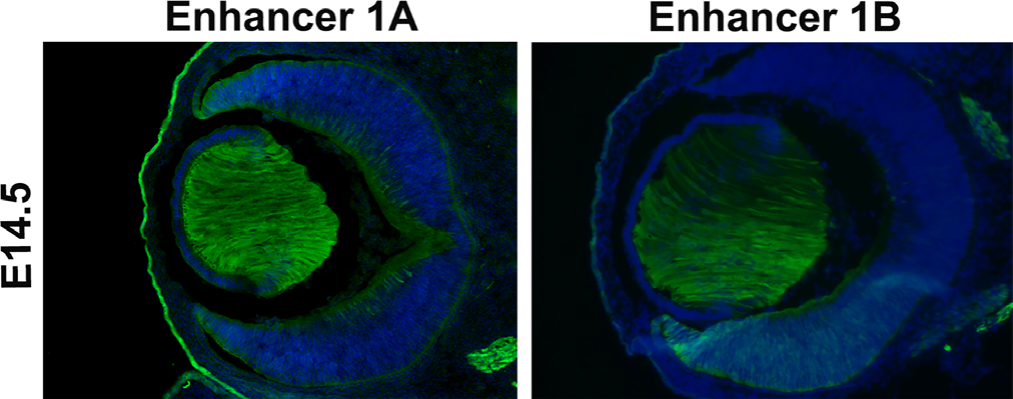
Analysis of separate 1A and 1B enhancers for Gata3 expression in embryonic lens. Delineation of eGFP expression for separate 1A and 1B enhancer regions in developing lens of the transgenic mice at E14.5. Enhancer 1A alone is sufficient for lens‐specific expression of the eGFP transgene. Enhancer 1B displays low asymmetric transgene expression in the developing lens.

To further characterize regions 1A and 1B and their in vivo activities, we cloned individual enhancers 1A and 1B as well as the 5‐kb Gata3 promoter in the parental peGFP‐1 vector and established additional transgenic mouse lines. We obtained four and three founders for the 1A and 1B lines, respectively. All enhancer 1A lines exhibited eGFP fluorescence in the eye during initial screening using the flash UV light (data not shown). Significantly, enhancer 1A alone was able to drive strong eGFP expression in the E14.5 lenses, with expression patterns very similar to that observed in 1AB embryos (Fig. 6). In contrast, region 1B was active only in one out of three lines, which showed weaker eGFP expression than animals harboring the joint 1AB enhancer. It is notable that expression of the 1B construct in this line was asymmetric in the lens, displaying higher eGFP levels at the temporal side of the elongating lens fibers (Fig. 6). Altogether, these data indicate that the individual enhancer region 1A is sufficient to drive Gata3 expression in the developing lens and that enhancer 1B region is likely to play a partially redundant role in this process.

### FGF2 and BMP4 Regulate Gata3 Promoter‐enhancer Reporters In Vitro

The cells that show the highest expression of Gata3 in the developing lens are in the posterior portion of the lens vesicle and subsequently form Gata3^+^ early lens fibers. Both cell populations are exposed to FGF and BMP from the vitreous humor, and both factors have been implicated in fiber differentiation (Lovicu and McAvoy, 2005). We therefore tested the Gata3 transcriptional elements in primary cultures of embryonic chick lens epithelial cells (DCDMLs), a well established serum‐free system that undergoes bona fide fiber cell differentiation in response to a 6‐day treatment with 15 ng/ml FGF2 and/or 10 ng/ml BMP (Le and Musil, 1998; Xie et al., 2016). To simplify the system, we initially tested enhancers 1AB, 1B, and 1A with a heterologous E4‐TATA promoter (Chauhan et al., 2004); however, no activity was detected in repeated experiments (data not shown). These results are in agreement with an earlier report demonstrating that some previously identified Gata3 regulatory elements are unable to direct the expression of a heterologous promoter in a tissue‐specific manner (Lieuw et al., 1997).

**Figure 7:**
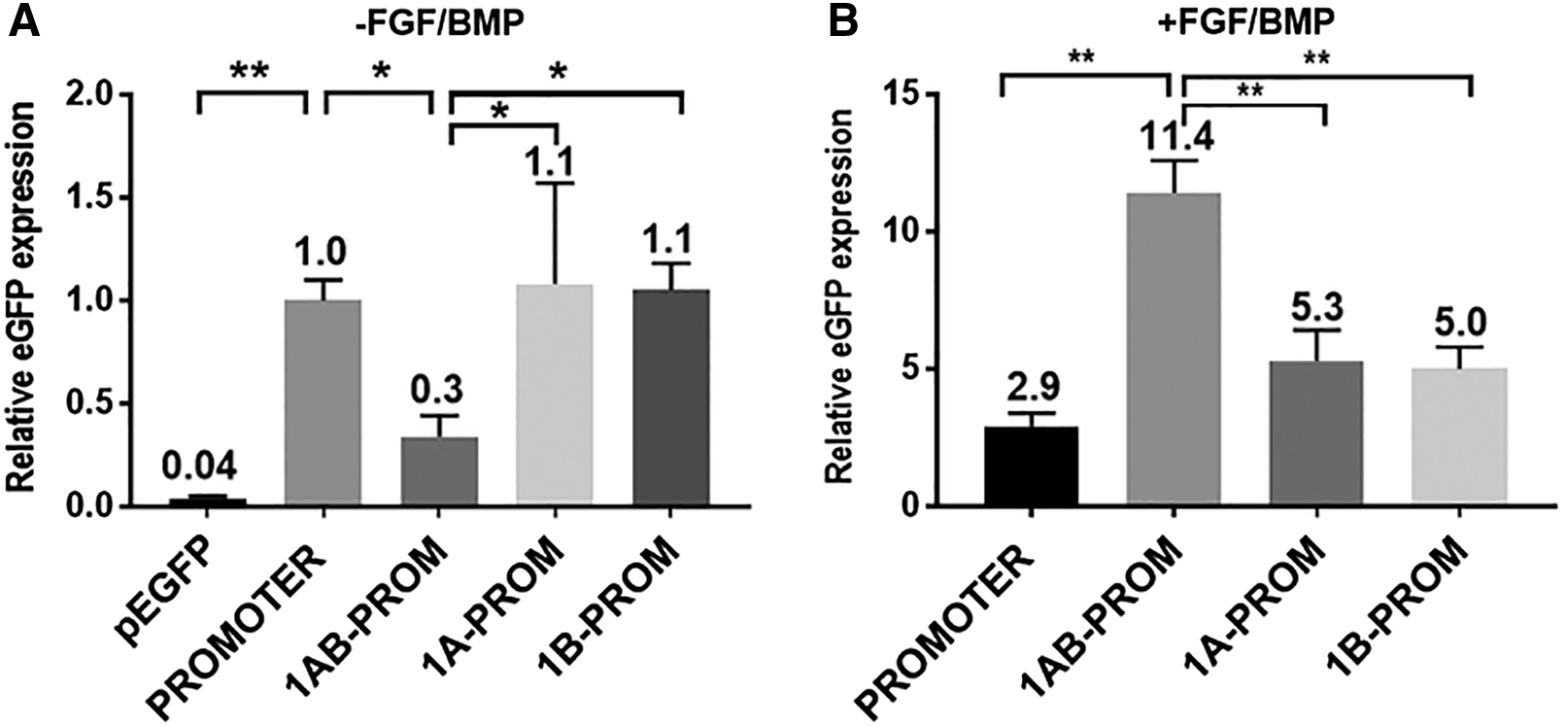
FGF‐ and BMP‐mediated regulation of lens‐specific Gata3 regulatory elements. **A**: Semiquantitative EGFP reporter expression analysis (Western blot, β‐actin used as loading control) after transient expression of PROM, 1AB‐PROM, 1A‐PROM, and 1B‐PROM constructs in primary cultures of embryonic chicken lens cells. The 5‐kb Gata3 promoter alone (PROM) is active in this system. **B**: Examination of FGF‐ and BMP‐regulated activity of Gata3 *cis*‐regulatory elements in primary chick lens cells. The ratio of GFP expression in cells cultured with BMP and FGF was quantitated as the fold change over expression of the same construct in the absence of added growth factors. Joint enhancer 1AB up‐regulates eGFP expression in response to FGF/BMP by ∼ 4‐fold compared to the PROM construct.

Thus, we next analyzed a series of enhancer‐promoter constructs used in transgenic experiments described earlier. Under basal (e.g., no added growth factor) conditions, expression of a 5‐kb GATA3 promoter‐peGFP construct was ∼25 times greater than that of maternal peGFP vector (Fig. 7A). Expression of a 1AB–5‐kb Gata3 promoter–eGFP construct was less (∼ 0.3‐fold) in these assays than that of the promoter‐only construct in 6/6 experiments. Expression of a 1B–5‐kb GATA3 promoter‐peGFP construct was comparable to that of 1A–5‐kb GATA3 promoter–eGFP (Fig. 7A). Next, we tested the ability of the reporter constructs to respond to fiber‐inducing levels of growth factors. Treatment with FGF and BMP increased the expression of 5‐kb Gata3 promoter–eGFP by an average of 2.9‐fold (Fig. 7B). Similar results were obtained with a 1B–5‐kb GATA3 promoter–eGFP construct (∼5X) and a 1A–5‐kb GATA3 promoter–eGFP construct (∼5.3X) (Fig. 7B). It is notable that addition of the 1AB element enhanced growth factor responsiveness to ∼11.4‐fold (Fig. 7B). Taken together, the results suggest that the 1A and 1B elements act in combination to confer enhanced growth factor responsiveness to the 5‐kB GATA3 promoter.

## Discussion

The present data provide novel insights into transcriptional regulation of Gata3 during mouse eye development through the identification of an evolutionarily conserved bipartite enhancer located ∼17–19 kb upstream from the TSS that is active in lens, cornea, and other ocular cells. Expression of EGFP driven by the 1AB construct in the lens recapitulates the endogenous pattern of Gata3 in mouse embryos, and studies of multiple reporters in differentiating lens cells support the idea that BMP and FGF signaling control expression of Gata3 in lens.

Our findings with Gata3^GFP^ mouse model and expression studies of multiple Gata3 enhancer constructs driving expression of eGFP proteins are consistent with previous studies of Gata3 expression during mouse lens development (Lakshmanan et al., 1999; Maeda et al., 2009). It is interesting to note that we observed Gata3 expression at E9.5 in the lens preplacodal region (Fig. 1B). In Zebrafish, Gata3 is expressed in the preplacodal ectoderm and directs its differentiation (Bhat et al., 2013; Yao et al., 2014). Gata3 was also observed in the prospective lens ectoderm and optic vesicle in chick (Sheng and Stern, 1999) and Zebrafish (Yao et al., 2014). We conclude that expression domains of Gata3 in the early anterior non‐neural ectoderm are evolutionarily conserved in vertebrate embryos. In mice, the requirement of Gata3 in global placodal development is still unclear and needs further investigation; nevertheless, inactivation of Gata3 by Pax6‐cre (Wolf et al., 2013) disrupts lens development after the formation of the lens vesicle (Martynova et al., unpublished data). Interestingly, Gata3 expression is down‐regulated after E16.5 and is not visible at E18.5 or in neonatal lens (data not shown), nor is it detectable by RNA‐seq data analysis (Zhao et al., 2018).

The 120‐kb YAC containing the *Gata3* locus was sufficient to confer eye‐specific expression in the developing eye (Lakshmanan et al., 1998; Lakshmanan et al., 1999). It is important to note that only one out of multiple tissue‐specific regulatory elements could drive Gata3 expression in vivo when linked to a heterologous promoter (Lieuw et al., 1997). These findings were confirmed by our pilot studies using the E4TATA heterologous promoter. We therefore examined Gata3 regulatory elements in conjunction with the 5‐kb Gata3 homologous promoter (‐4.3/+0.6 kb). The eGFP reporter assays in transgenic mice demonstrated that a 1AB construct recapitulated endogenous Gata3 expression in the developing lens with additional activity in the cornea and retina (Fig. 3B). Our analysis, as well as earlier studies of Gata3, did not reveal expression in the cornea and retina (Lakshmanan et al., 1999; Quina et al., 2005; Debacker et al., 1999). The observed differences in reporter eGFP and endogenous Gata3 expression domains are likely caused by the absence of one or more negative regulatory elements for these tissues. Enhancers 1D and 7 displayed transgene expressions in ocular muscles and in the neighboring ectodermal tissues, but not in the developing lenses (Fig. 3B). Indeed, Gata3 plays a significant role in face morphogenesis, as germ line mutation of Gata3 exhibited prominent craniofacial abnormalities (Pandolfi et al., 1995). It is interesting to note that the 5‐kb Gata3 promoter alone controlled transgene expression in the facial skeleton (data not shown). This observation is in agreement with previous data showing that a DNA region ‐2052/‐308 bp is necessary for Gata3 regulation in cranial ganglion cells (Lieuw et al., 1997). Gata3 is also indispensable for normal inner ear development (Karis et al., 2001; Lawoko‐Kerali et al., 2002; Duncan and Fritzsch, 2013). Gata3 haploinsufficiency causes human hypoparathyroidism, sensorineural deafness, and renal dysplasia (HDR) syndrome (Van Esch et al., 2000). An earlier study identified a ‐2052/+1004 bp region in the mouse locus that contains an element for Gata3 expression in the inner ear (George et al., 1994). We also observed transgene expression in the developing ear using the 5‐kb promoter construct (data not shown), as well as low activity in the lens vesicle and differentiating lens fiber cells (Fig. 3B). It is important to note that the addition of 1A and 1B elements dramatically augments eGFP fluorescence in the developing lens (Fig. 3B). We conclude that enhancer 1AB in conjunction with the 5‐kb Gata3 promoter drives transgene expression in the differentiating primary and secondary lens fiber cells, but not in the proliferating lens epithelial cells at the anterior part of the lens (Fig. 3B).

It is now established that enhancer regions are enriched with clusters of transcription factor recognition motifs for local activators and SRTFs (Barolo and Posakony, 2002; Long et al., 2016). These combinations ensure integration of intrinsic and extrinsic signals to precisely regulate gene expression. Cell cycle exit‐coupled differentiation of primary lens fiber cells is tightly regulated by FGF, BMP, and Notch signaling (reviewed in Cvekl and Ashery‐Padan, 2014; Lovicu et al., 2011). Differentiation of primary lens fiber cells requires cooperation between BMP and FGF signaling (Jarrin et al., 2012). BMP controls both Gata3 expression in the hair follicles (Kobielak et al., 2003; Genander et al., 2014) and cranial neural crest cells (Bonilla‐Claudio et al., 2012). GATA3 is a bona fide effector functioning downstream of Notch signaling in non‐lens systems (Fang et al., 2007; Sakata‐Yanagimoto et al., 2008), and Notch signaling regulates cell cycle exit in the lens (Le et al., 2009; Saravanamuthu et al., 2012; Jia et al., 2007; Rowan et al., 2008). We found that the Gata3 promoter contains clusters of FGF‐regulated AP‐1 factor motifs and BMP‐responsive Smad sites. Enhancer 1AB also includes multiple Gata3 sites (Fig. 4). The Gata3 autoregulatory mechanisms have been described elsewhere (Ouyang et al., 2000; Lee et al., 2000; Scheinman and Avni, 2009). In addition, enhancer 1A contains putative binding motifs for Prox1 and Pitx3 (Fig. 4A). Prox1 plays a crucial role in lens fiber differentiation (Wigle et al., 1999), and exogenous FGF is sufficient to up‐regulate expression Prox1 levels in lens explants (Audette et al., 2016). Furthermore, deletion of FGF receptors leads to aberrant Prox1 expression in the lens (Zhao et al., 2008). A putative binding motif for Pitx3 was localized in the enhancer 1A. Pitx3 is expressed in the lens vesicle and is later limited to lens epithelium (Shi et al., 2006; Ho et al., 2009), and Pitx3 deletion causes apoptosis, abnormal lens fiber differentiation, and loss of the lens (*aphakia)* (Semina et al., 1997; Rieger et al., 2001; Wada et al., 2014). In the Pitx3 knockout lens, Prox1 expression is observed throughout the entire lens (Ho et al., 2009). We propose that Prox1 serves as an activator and Pitx3 as a repressor of Gata3 transcription in the lens.

Our earlier studies established a link between FGF signaling and crystallin expression mediated via AP‐1 (c‐Jun) and Etv5/ERM (Xie et al., 2016). Enhancer 1A is highly enriched in AP‐1 binding motifs, indicating potential interactions between FGF‐regulated transcription factors and Gata3 expression. Multiple Smad binding sites are predicted within enhancer 1B. Additional Smad‐binding elements located in the evolutionarily conserved 5’‐flanking regions of the Gata3 promoter have previously been identified elsewhere (Bonilla‐Claudio et al., 2012). A binding site for Notch signaling transcription factor Rbpj was found within the Gata3 promoter elsewhere (Amsen et al., 2007). It is possible that non‐canonical Smad‐binding sites exist in both 1A and 1B sequences, as shown in other systems when Smads partner with CBP, c‐Fos, JunB, and other proteins (Ampuja and Kallioniemi, 2017).

To narrow down the regulatory element that drives Gata3 expression in the lens, we separated enhancers 1A and 1B and analyzed them individually in transgenic mice. We found that enhancer 1A alone exhibited strong eGFP fluorescence in the lens, whereas eGFP expression in the lens driven by enhancer 1B was weaker (Fig. 6). It is notable that expression under the control of the 1B element was asymmetric, a pattern reminiscent of previous observations that early differentiation of primary lens fiber cells occurs on the temporal side of the lens vesicle and is followed by delayed differentiation on the nasal side (Faber et al., 2002).

While enhancer 1AB coupled to the Gata3 promoter was indispensable for lens‐specific expression of Gata3, we observed that the Gata3 promoter itself was sufficient to drive the FGF‐/BMP‐stimulated expression of the reporter gene in the primary embryonic chick lens system (Fig. 7). This observation is consistent with similar studies of c‐Maf expression in vivo and using the in vitro DCDMLs. The 1.3‐kb c‐Maf promoter requires its distal lens‐specific enhancer in mice in vivo (Xie and Cvekl, 2011). However, the c‐Maf promoter alone is up‐regulated by FGF signaling in vitro (Xie et al., 2016). In addition, our preliminary studies indicate that both individual enhancer regions 1A and 1B coupled to the 5‐kb Gata3 promoter are activated by BMP4 and FGF2; however, like with the 1AB‐promoter system (Fig. 7), no synergistic effects were observed. Thus, additional experiments are needed to dissect the *cis*‐regulatory wiring of enhancers 1A and 1B and Gata3 promoter. One of the possible long‐term outcomes of these studies is a development on new Cre‐lines for regionally specific gene targeting in the lens.

In summary, we have identified and characterized a novel lens‐specific Gata3 bipartite enhancer that lies 18 kb upstream of the Gata3 structural gene. This enhancer is regulated by FGF and BMP signaling in vitro. The present findings thus integrate Gata3 into the gene regulatory networks regulated by FGF and BMP signaling during mouse lens development, including transcription factors c‐Maf and Prox1. Further studies will be needed to examine the role of individual transcription factors that regulate transcription of Gata3 through enhancer regions 1A and 1B. To address necessity of the bipartite eye‐specific enhancer, deletions of 1A, 1B, and 1AB regions in mouse oocytes using the CRISPR system will be required, followed by analysis of mouse eye development.

## Experimental Procedures

### Reporter Plasmids and Transient Transfections

Gata3 putative enhancer regions were generated by PCR from Genomic Clone RPC B731M06136Q (Source BioScience, UK) using Phusion High‐Fidelity PCR Master Mix (New England BioLabs, USA). The purified fragments were subcloned into pGL3 vector (Promega, USA) fused to a minimal 4ETATA promoter (Chauhan et al., 2004). The primers for PCR amplification and region locations for each DNA fragment are listed in Table 2. Transient transfections were performed in human embryonic kidney 293T cells using Lipofectamine 2000 (Thermo Fisher Scientific, USA). Briefly, 0.8 µg of the reporter gene and 20 ng of Renilla‐TK were contransfected into the cells in 24‐well plates. The cells were harvested 48 hr post‐transfection, and the firefly and Renilla luciferase enzyme assays were conducted using the dual‐luciferase reporter assay system (Promega, USA). The firefly luciferase enzyme activity was normalized to Renilla luciferase enzyme activity.

**Table 2:** Primers for Cloning and Location of Candidate Gata3 Enhancer Regions for in vitro Testing in Dual‐luciferase Reporter Assay

Region	Forward primer	Reverse primer	Size, bp	Chromosome location	Location relative to Gata3 TSS. kb
E1A	GAAAAGAGGTGTGGGTCGAG	GCTGAAGACTGGTGCCAAG	615	chr2:9,897,700–9,898,314	−19.1/–19.7
E1B	AGTGGGGGAGGGTACAGAGA	CTGAGTGATTCCCACCATCC	657	chr2:9,895,741–9,896,397	−17.1/–17.8
E1C	CCACCAGTTCTTGTCCTTCC	GACTCTCCCCCTTCCTGAAT	714	chr2:9,894,706–9,895,419	−16.1/–16.8
E1D	AGAACCCTCAGACCCAATCC	CTGGGGAGAGAGGGACTCTT	867	chr2:9,892,404–9,893,270	−13.8/–14.7
E2	CCTCCCTTGGCTCAGTGTAG	TTCTCCCAGGAGTTGACCAC	1164	chr2:9,887,812–9,888,975	−9.2/–10.4
E3B	AGAGAGGTGCCTGCTACGTG	CCCCTTTATTCCTCCGTGTC	767	chr2:9,877,118–9,877,884	+0.7/+1.3
E4A	TGTCCAAGCCCCATACTCTC	CTGCTCAGGTCTCCCTTCTC	443	chr2:9,875,853–9,876,295	+2.3/+2.7
E4B	GCTTACCTGTGCTGGATCGT	GTCTCAGGGCAGCTCTCACT	642	chr2:9,874,383–9,875,024	+3.6/+4.2
E5	ACGAGGCTACCTCCTTCTCC	CTGCTGAGGGACTTCTGGAT	1188	chr2:9,872,892–9,874,079	+4.5/+5.7
E6	GAACCAGCTCCCCTTTTAGG	GCCTCTGCTTCTCAAGTGCT	975	chr2:9,852,750–9,853,724	+24.9/+25.9
E7	CAGGCCCTCAAGTATGTTGG	ACCCTGGCCTGTAGACTGAG	757	chr2:9,835,127–9,835,883	−42.7/–43.5
E8	AGGGTATGTGTGCCCTCTTG	GAGCTGGAATGGGAAGTGAC	835	chr2:9,881,939–9,882,773	−3.3/–4.2

### Generation of Transgenic Mice

Animal husbandry and experiments were conducted in accordance with the approved protocol of the Albert Einstein College of Medicine Animal Institute Committee and the ARVO Statement for the Use of Animals in Ophthalmic and Vision Research. Three reporter plasmids were generated in peGFP‐1 (Clontech, USA) as schematically depicted in Figure 3A. The transgenes were released from the vectors by AfeI and DraIII digestion. Transgenic mice were generated by pronuclear injection of the fertilized eggs at the AECOM Transgenic Core Facility. Briefly, female FVB mice are superovulated with pregnant mare serum (PMS) and human chorionic gonadotropin (hCG) ∼72 and ∼24 hr (respectively) prior to date of pronuclear injection. After hCG, matings are set up using the superovulated females and FVB males. The following morning, mating plugs are checked and only females with plugs are euthanized and have their oviducts excised. Oviducts are dissected and zygotes isolated. Zygotes are pronuclear injected with a DNA construct and then transferred into a CD1 pseudopregnant. Pups are born 20 days after implantation. Initial genotyping of the mice was performed using BlueStar Flashlight and barrier filter glasses (Nightsea, USA) followed by semiquantitative PCR using primer (5’‐AGCTTGCGAAGACCTAGTGC‐3’ and 5’‐ GAACTTCAGGGTCAGCTTGC‐3’) that spans over promoter‐eGFP region. Gata3‐eGFP mouse line was described previously (Grote et al., 2006). The primers for eGFP genotyping (5′‐ACCCTCGTGACCACCCTGACCTAC‐3′, 5′‐GACCATGTGATCGCGCTTCTCGTT‐3′) are described elsewhere (Barrow et al., 2005).

### Analysis of GFP Expression by Immunofluorescence

Staged embryos were fixed in 4% paraformaldehyde overnight, cryoprotected in 30% sucrose in phosphate‐buffered saline, and embedded in optimal cutting temperature Tissue Freezing Medium (Triangle Biomedical Sciences, USA) for cryosectioning; 10‐μm transverse sections were collected, washed in PBS, and incubated for 30 min with Image iT FX Signal Enhancer (Molecular Probes, USA). Slides were washed in PBS and incubated overnight at 4°C with rabbit anti‐GFP (1:2000) (Invitrogen, USA; A‐11122). Sections then were washed three times for 10 min in PBS and incubated for 2 hr with the secondary antibody, goat anti‐rabbit Alexa Fluor 488 (1:200) (Molecular Probes, USA). Cell nuclei were counterstained with DAPI (1:1000) for 10 min (Sigma, USA). Slides were washed with PBS and mounted with Vectashield (Vector Labs, USA). Images were taken with ZEISS Axio Observer fluorescent microscope (ZEISS, Germany). Relative eGFP expression was measured as fluorescence intensity from three different sections using ImageJ software. eGFP staining of wild‐type lens was set as a reference point.

### Quantitative PCR for Transgene Copy Numbers

Genomic DNA (gDNA) from transgenic animals was extracted by overnight digestion of the tail in 100 µl of DNA digestion buffer (50 mM Tris‐HCL pH 8.0, 100 mM EDTA pH 8.0, 100 mM NaCl, 1% SDS) with addition of proteinase K to final concentration of 0.5 mg/ml. Proteinase K was inactivated by incubation of the digested tail at 95°C for 15 min. DNA was precipitated by addition 100 µl of isopropanol, washed with 70% ethanol, air‐dried, and resuspended in 100 µl of nuclease‐free water. gDNA concentration was measured by NanoDrop ND‐2000 (Thermo Fisher Scientific, USA), and each sample was diluted to 5 ng/µl; 10 ng of gDNA was used for real‐time PCR reaction in each well of a 96‐well plate. Number of transgene integrations was measured by TaqMan qPCR in Universal PCR Master Mix using EGFP TaqMan assay (Mr00660654_cn) according to manufacturer's protocol (Thermo Fisher Scientific, USA). Quantitation of eGFP copy number was normalized on endogenous mouse Tfrc reference gene (Thermo Fisher Scientific, USA; 4458367).

### Bioinformatics Analysis

Most of transcription factor binding sites were retrieved from the JASPAR database (Bryne et al., 2008); putative Smad‐sites were predicted by using Smad consensus motifs 5′‐GTCTAGAC‐3’ (Shi et al., 1998) and 5′‐CWGSMGCY‐3’ (Morikawa et al., 2011). The AP‐1 and Ets motifs were obtained from the previous studies (Wei et al., 2010; Li et al., 2011; Yang and Cvekl, 2007). A RBPJ consensus motif 5’‐GTGRGAA‐3’ was described elsewhere (Wang et al., 2011; Castel et al., 2013). Comparative sequences analysis for evolutionary conservation was performed by using VISTA Point tool (Frazer et al., 2004). The motif search was performed using Fuzznuc software.

### Primary Chick Lens Cell Cultures, Transfections, and Western Blotting

Dissociated cell‐derived monolayer lens cultures (DCDMLs) were prepared from E10 chicken embryos as described previously (Le and Musil, 1998). The cells were plated at 1.0 x10^5^ cells/well density in laminin‐coated 96‐well tissue culture plates in M199 plus BOTS (2.5 mg/ml bovine serum albumin, 25 µg/ml ovotransferrin, 30 nM selenium) with penicillin G and streptomycin. The day after plating, DCDML cultures were transfected using Lipofectamine 2000 (Thermo Fisher Scientific, USA) and treated 5 hr later with 15 ng/ml FGF‐2 and 10 ng/ml BMP‐4 (R&D Systems, USA). The cells were lysed 6 days later in SDS‐PAGE sample buffer, and equal amounts of lysate were transferred to PVDF membranes. The blots were probed with primary anti‐GFP antibody (Clontech, USA) and secondary antibody conjugated to Alexa Fluor 680 (Molecular Probes, USA). The staining was analyzed by the Odyssey infrared imaging system (LI‐COR Biosciences, USA). The results of eGFP immunoreactivity were normalized to β‐actin staining in the same sample.
